# Smart Biomaterials Shaping the Future of Dentistry: A Comprehensive Review

**DOI:** 10.7759/cureus.101904

**Published:** 2026-01-20

**Authors:** Jyotsna Sethumadhavan, Munazzeh Fakhrealam Siddiqui, Prachi Gholap, Sheetal M Jadhav, Nilesha Vilasrao Kadam, Saudamini More

**Affiliations:** 1 Prosthodontics, Crown and Bridge, Bharati Vidyapeeth (Deemed to be University) Dental College and Hospital, Navi Mumbai, IND; 2 Public Health Dentistry, Bharati Vidyapeeth (Deemed to be University) Dental College and Hospital, Navi Mumbai, IND

**Keywords:** biocompatibility, durability, shape memory, smart behaviour, toxicity

## Abstract

Dentistry revolves around the diverse dental materials being used in each and every step for a successful outcome of the treatment. Traditional materials require a specific design to meet performance requirements for load, speed, and lifespan expectancy. They seem difficult to modify their specifications or respond to changes in the environment. Traditional materials pose numerous disadvantages, such as limited durability and potential toxicity. As a result, smart materials were introduced where they respond to external stimuli, including stress, pH, temperature, moisture, and magnetic and electric fields. In the 1970s and 80s, smart materials were introduced in the market, with the first applications utilizing magnetostrictive technology, specifically nickel-titanium alloys that possess shape memory properties. In the 1980s, these materials paved their way into the field of dentistry, but their importance and applications flourished in the 20th century. A key feature of smart materials is their ability to return to their original state even after the stimulus has been removed, making them ideal for use in dentistry. A few examples include orthodontic shape-memory alloys, smart sutures, and resin-modified glass ionomer cement (GIC). This review aims to understand the latest advancements in smart dental materials and assess their benefits over traditional materials. The integration of smart materials improves treatment effectiveness and longevity, allowing for minimally invasive approaches. Smart materials enhance patient comfort and improve material performance.

## Introduction and background

McCabe defined "smart materials" as materials whose properties may be altered in a controlled fashion by stimuli, such as stress, temperature, moisture, pH, and electric or magnetic fields [1]. Materials that are able to alter their properties in response to external stimuli are smart materials or can be called responsive materials, as they adapt to environmental changes, such as changes in temperature, pressure, etc. These materials have been used in the USA since the 1980s, where they were termed smart/intelligent materials [1].

In contrast, materials intended for long-term applications within the body or oral cavity are generally passive, meaning they are designed to remain stable without interacting with their surroundings. They are known as biomimetic materials, as they mimic the enamel and dentin components of the tooth structure. The term "biomimetics" was coined by Otto Schmitt in 1950, which refers to the study of the design of materials and their engineering based on their biological structures and functions [2]. Smart materials are also termed "responsive materials" due to their behavior of responding to external stimuli [3].

**Figure 1:**
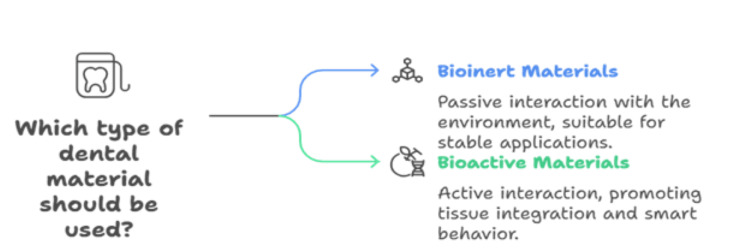
Types of smart materials Image created by the authors.

Smart behavior of a material depends on its ability to be able to change in response to the surroundings in an effective, consistent, and reversible way. Shape-memory alloys and polymers are some examples that can change in response to temperature. As research continues, we can see more groundbreaking inventions that can transform this traditional environment. Depending on their interaction with the environment, they can be classified as bioinert or bioactive (Figure 1). Today, the most promising technologies for lifetime efficiency and improved reliability include the use of "bioactive" smart materials [4].

In dentistry, the need for advancement in dental materials has become essential; materials need to be chemically stable, biocompatible, and nonreactive in the oral cavity so that they do not harm the surrounding healthy tooth structure. Many such materials are already in clinical use in today's modern dentistry.

History

**Figure 2:**
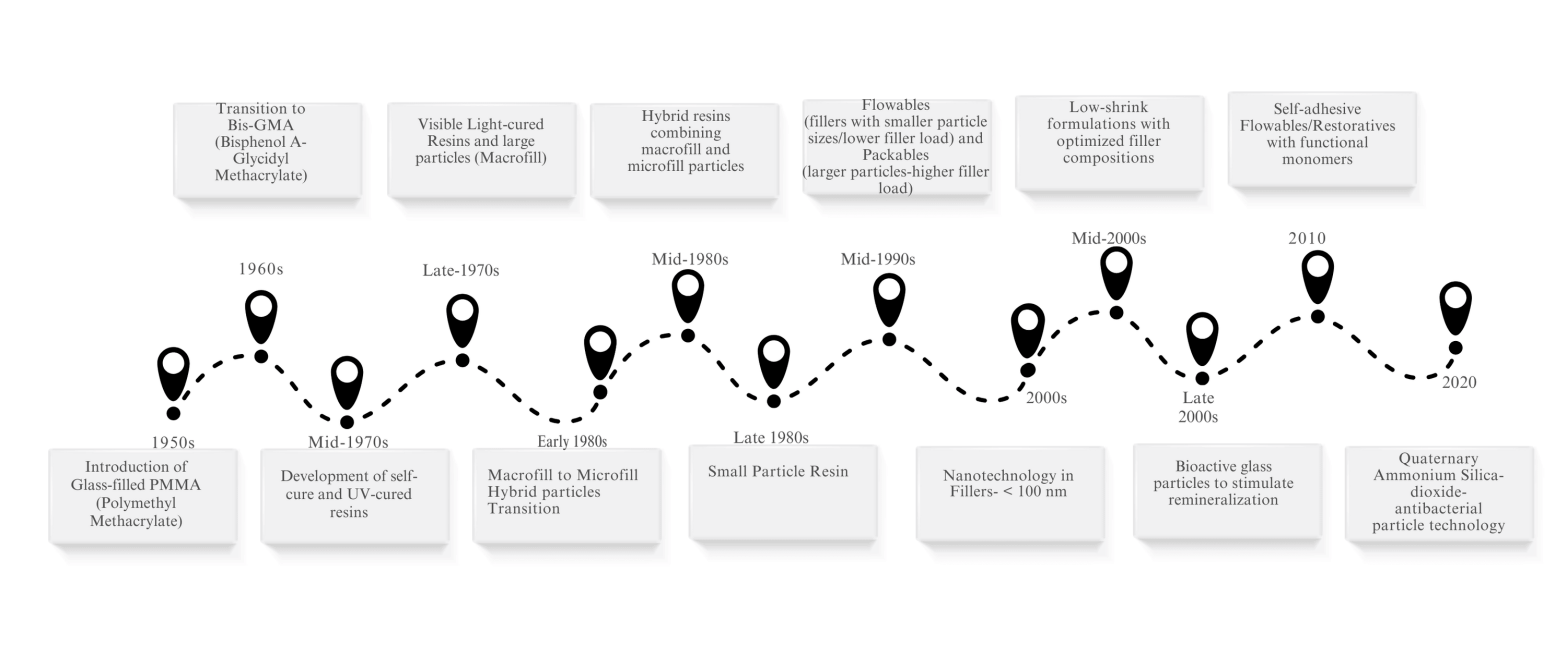
History of smart materials Image created by the authors.

Smart materials were minimally started from being introduced in the 1950s and are experiencing ongoing advancements to ensure a predictable outcome (Figure 2) [5].

## Review

Classification

**Figure 3:**
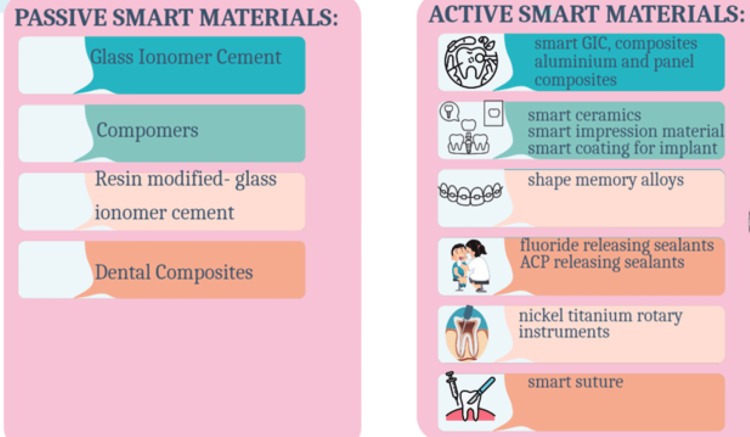
Examples of passive and active smart materials ACP: Amorphous calcium phosphate. Image created by the authors.

The smart materials can be broadly classified as (1) Passive smart materials: materials that continuously respond to environmental changes or stimuli but do not require an external etiological factor to change their characteristics significantly. They are able to self-repair. (2) Active smart materials: these materials respond positively to changes in the external stimuli or when additional material support is required. They repair once an external stimulus is present in the environment (Figure 3) [2].

Properties

**Figure 4:**
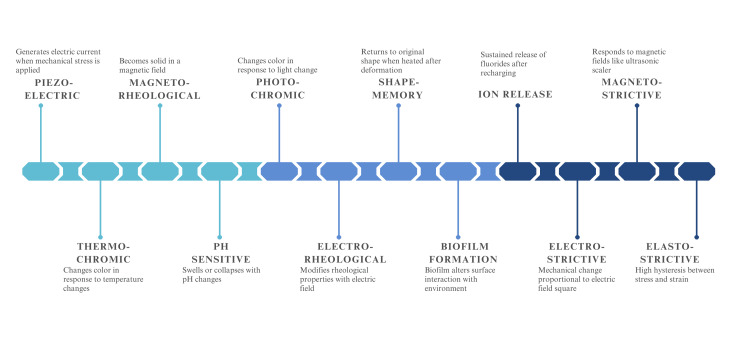
Properties of smart materials Image created by the authors.

Smart materials are environmentally sensitive and adapt themselves accordingly. Stayton et al. designed biological delivery systems that are reversible and nontoxic. The delivery systems had the ability to change their structural and functional properties, thereby demonstrating remarkable smart material properties [7]. The different properties of smart materials are summarized in Figure 4 [2,6].

Implications

Smart materials have been widely accepted and incorporated to achieve successful treatment outcomes. Rolland et al. hypothesized that an antibacterial dentine-bonding resin containing methacryloxydodecyl-pyridiniumbromide (MDPB) may reduce biofilm formation [8]. The study supported earlier studies that indicated a composite containing an MDPB-containing filler showed reduced bacterial adhesion and glucan synthesis [9]. Antimicrobial, anti-degradation, and biocompatibility are the key properties leading to a favorable accomplishment in the usage of such intelligent materials [10]. These materials possess varied functional implications, such as preventing caries, improving marginal integrity, reducing wear, and extending longevity in all dental fields.

Smart materials

Smart Glass Ionomer Cement (GIC)

GICs are the most widely used cements in the dental practice. They absorb quickly and release a solvent that creates a gel-like structure that is similar to human dentin. The ability of movement of water in and out of the structure of GIC in response to the dimensional change and heat alteration is called the coefficient of thermal expansion. This is the reason why GIC shows better marginal adaptation, but bacterial adhesion is still a concern, which causes secondary dental caries [11]. It can be used as liners, bases, and reinforce restorative materials.

Fluoride-releasing dental restorative materials have the following effects: Fluoride has an antimicrobial effect, and it converts hydroxyapatite crystals to fluorapatite, which is more acid-resistant [12]. Burst effect, i.e., initial high release of fluorides and further sustained release over a period of time, which we can replenish again by using fluoride-based toothpaste or topical fluoride (i.e., topping-up effect).

Smart composites

Smart composites are ion-releasing materials that release fluoride, calcium, and hydroxyl ions at the critical pH, i.e., 5.5. When the critical pH drops, the smart composites start to neutralize the acid. The fluoride release from composites is lower than that from GIC but higher than that from compomers.

More modified smart composites contain amorphous calcium phosphate (ACP), an antecedent of hydroxyapatite (HAP) with both restorative and preventive properties, which can be used as a pit-and-fissure sealant, adhesive, and dental cement [12].

When the pH drops below the critical level, the ions merge to form a gel matrix, which later becomes amorphous crystals of calcium and phosphate ions, causing remineralization. Here, calcium causes remineralization after becoming supersaturated, while phosphates neutralize the acid through buffering (e.g., Ariston pH control, introduced in 1998 by Ivoclar-Vivadent).

Casein Phosphopeptide (CCP) and ACP

CCP and ACP help in the remineralization process by replacing the essential minerals lost from the tooth through assistance by saliva. The CPP binds to the plaque surrounding the tooth, thus presenting a soluble CaPO₄ at high concentration. While in amorphous form, CaPO₄ can penetrate tooth enamel, and the remineralization process occurs (e.g., Recaldent).

Self-healing composites

Composites fixed using a self-repairing process formulated on microcapsules might be effective for macroscopic repair techniques. It contains healing powder and liquid encapsulated by silica nanoparticles, which increases the bond strength of the resin and reduces fracture. It uses a formulation of bisphenol A-glycidyl methacrylate at 45% wt/wt, with 0.7 wt% silane glass included, encapsulated dicyclopentadiene (microcapsules), and a Grubbs catalyst (a transition-metal carbene complex using ruthenium), which heals microcracks [13].

Smart dentin replacement

Dentsply introduced posterior composites for class 1 and class 2 cavities due to their flowable characteristics and low polymerization shrinkage. It can be applied in increments up to 4 mm. It is chemically compatible with all methacrylate-based composites and posterior composites and is used to replace the enamel occlusal layer and complete an adhesive restoration that improves marginal adaptation. Bulk-fill composite resin is used in both incremental and bulk-fill techniques to overcome the disadvantages of incremental methods [14].

Smart impression materials

Irreversible hydrocolloids, such as alginate, and elastomeric materials, such as polyvinyl siloxanes and polyethers, are the standard dental materials used for taking impressions. They are modified to exhibit shape-memory properties, preventing distortion of impressions. These possess thixotropic properties along with being hydrophilic to make void-free impressions [12]. The snap-set properties of these materials allow minimal distortion while reducing working and setting time by 33% [15] (e.g., Imprint 3 VPS, Impregum, and Aquasil Ultra (3M ESPE)).

Smart burs

Smart burs are made up of polyether-ketone-ketone. The polymer was created to be softer than the healthy dentin and harder than carious (infected) dentin. This allows selective removal of decayed tissues and prevents overcutting of the healthy part, which has the ability to remineralize. When a smart bur comes in contact with a healthy structure, the bur becomes dull and vibrates [12,16].

**Figure 5:**
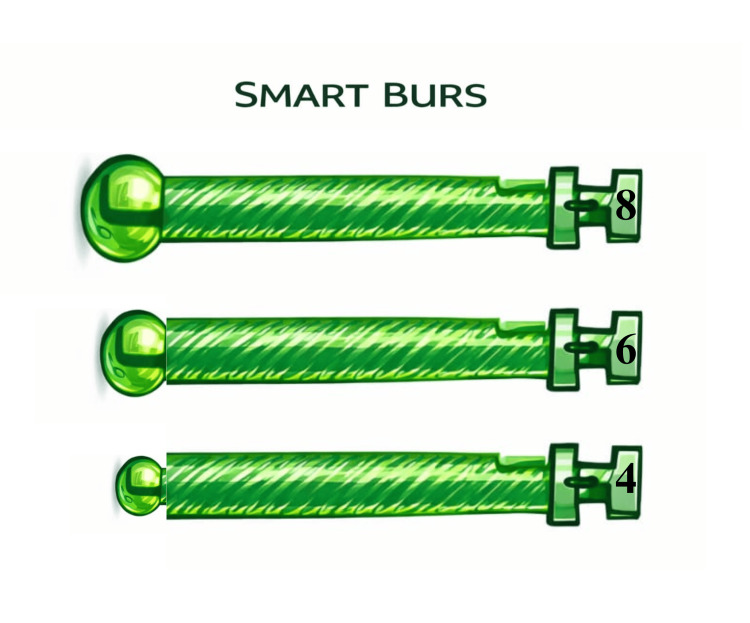
Smart burs Image Credit: Schematic representation designed by Dr. Munazzeh Fakhrealam Siddiqui.

The following is the cutting efficiency of the materials: for smart prep burs = 50 kH, carbide burs = 1600-1800 kH, infected dentin = 0- 30 kH, healthy dentin = 70-90 kH, and enamel = 360-430 kH. The standardized sizes are 010, 014, and 018 (self-limiting action) (e.g., SS White Diamond and Carbide preparation kit) (Figure 5).

Smart ceramics

ETH Zurich developed the first ceramic tooth bridge in 1995. The zirconia-based ceramic material being created was a single, metal-free structure rather than baked on different layers. The result is a biocompatible, high-strength restorative material resistant to cracking [17].

Unlike alumina, zirconium oxide transforms into different crystalline forms during the process of firing. At 950°C, i.e., during the firing process, it is tetragonal in structure while being monoclinic at room temperature, which occupies 4.4% more volume. When stress develops near the tip of the growing crack, the volume increase becomes a benefit by modifying the material's structure around the crack, which induces closure stress and halts its propagation. The stress-induced crystallographic transformation makes zirconia a smart material (e.g., Cercon, Cerec).

Smart diamond wedge

**Figure 6:**
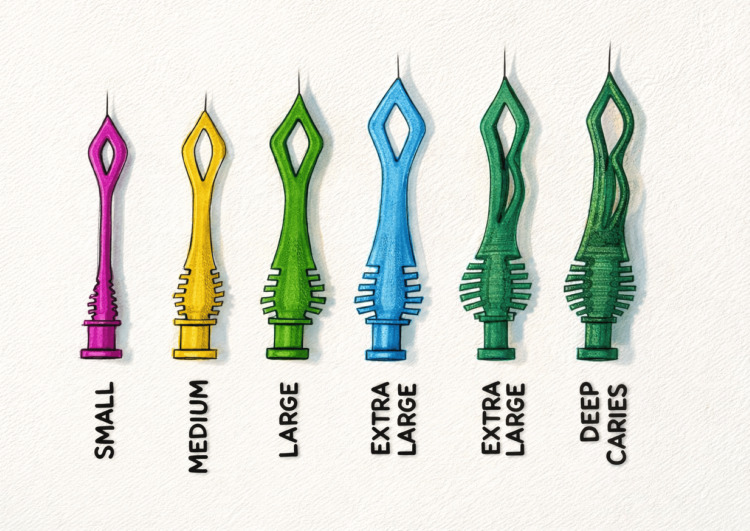
Smart diamond wedges Image Credit: Schematic representation designed by Dr. Jyotsna Sethumadhavan.

The wedge is a diamond-shaped cut at the fontanel, which decreases insertion pressure by collapsing during placement (Figure 6). The embrasure prevents slipping out through the spring-back opening. The entire structure provides a complete marginal seal, prevents excess flow, and eliminates the need for cleanup after the composite sets (e.g., Palodent).

Smart sutures

Sutures are composed of thermoplastic polymers that are biodegradable and capable of shape memory. They are temporarily applied loosely, with the ends secured. When the temperature exceeds the thermal transition point, the suture begins to tighten. Some sutures are synthetic or can be natural, and they can be embedded with temperature sensors that can detect infections. Current advancements aim to incorporate suture, which can accelerate wound healing, and electronic threads containing drug-infused polymers that exhibit therapeutic properties in response to heat and electrical stimuli [6] (e.g., a Novel MIT polymer).

Smart antimicrobial peptides

**Figure 7:**
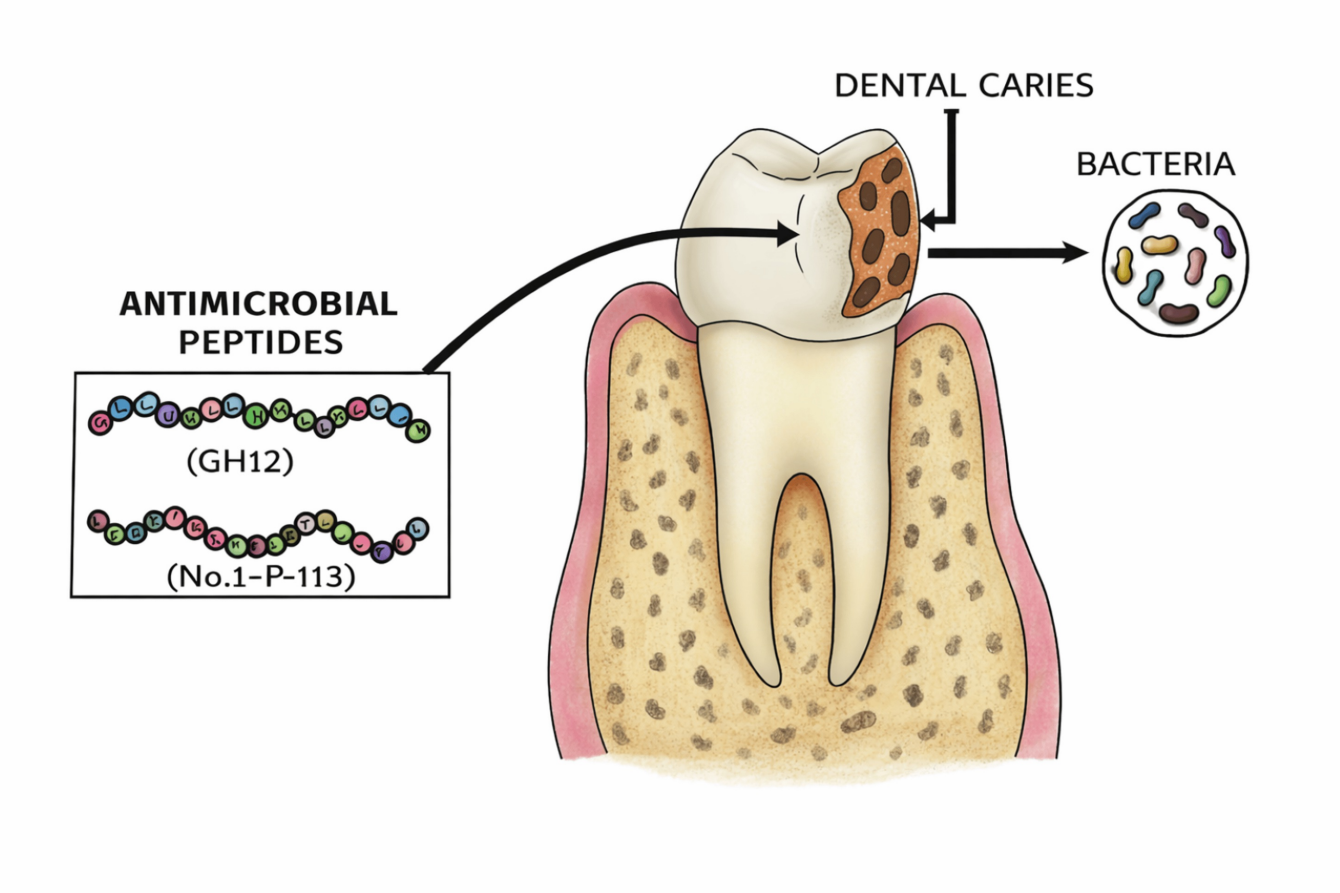
Smart antimicrobial peptide Image Credit: Schematic representation designed by Dr. Jyotsna Sethumadhavan.

These antimicrobial peptides are targeted to kill *Streptococcus* *mutans*, which are responsible for causing dental caries (Figure 7). The idea of tissue regeneration is that dental tissues can be regenerated; in general, BRAX-1 is thought to be responsible for enamel regeneration. By utilizing competence-stimulating peptides (CSP), a pheromone is created by *S. mutans* that removes the bacterial biofilms without disturbing the oral flora. These have the potential for selective killing and thus are known as selective probiotic antibiotics [18]. STAMPs are based on the fusion of a wide-spectrum antimicrobial peptide domain with a species-specific targeting peptide domain [19] (e.g., pheromone-guided "smart" antimicrobial peptides).

Smart coating for dental implants

These were primarily used in surgical implants for the hip and knee to enhance bone bonding and decrease the risk of infection. Over the period of time, the amorphous layer disintegrates and releases calcium and phosphates that help in bone growth. Future research can lead to the incorporation of silver nanoparticles that provide protection at implant sites from any infection. As the silver dissolves, the amorphous layer forms more rapidly, leading to rapid healing in the patient [20] (e.g., SGS Dental Implant Surface Treatment Smart Bioactive Trabecular Coating (SBTC)).

Smart fibers for lasers

Laser radiation of high fluency is transmitted through the body using photonics crystal fibers, which are compatible with 1.06 micrometer laser radiation, while YAG fibers of 14 micrometer core focus on enamel ablation. These are used in detection and optical diagnosis [21] (e.g., hollow-core photonic crystal fibers (PCFs)).

Smart memory alloys

NiTi is 56% wt nickel and 44% wt titanium; it has three phases: austenite, martensite, and R phase [21]. At room temperature, austenite has a body-centered cubic structure, and on cooling, it transforms to martensite, which is monoclinic due to thermal expansion. The R phase, or the mixed phase, occurs during a temperature change, modifying the alloy's properties [22]. Al-Horini et al. concluded that the temperature had a significant effect on the mechanical behavior of all test NiTi wires; the superelastic type behaved similarly to the thermal wires [23]. NiTi alloys are used in endodontic files and orthodontic wires. It also decreases the postoperative pain in patients while providing easier and faster accessibility for the dentist. These materials possess numerous advantages, such as (1) increased flexibility: reducing the risk of procedural errors and improving treatment outcomes; (2) shorter treatment time: streamlining procedures for greater efficiency; (3) reduced errors: minimizing zipping, ledges, and transportation issues; (4) shape-memory effect: our instruments can change shape in response to temperature, ensuring optimal performance [24]; and (5) corrosion resistance: a protective titanium oxide layer shields our instruments from corrosive environments [25].

Smartpaste bio

Smartpaste bio contains bioceramics that give calcium hydroxide and hydroxyapatite as byproducts, giving it bactericidal and bacteriostatic properties. It is used as a root canal sealant and requires 4-10 hours of setting time. Here, the hydrophilic component, i.e., calcium hydroxide, absorbs moisture and swells up, filling the empty spaces. This provides dimensional stability in root canal treatment [13].

Smartseal obturation system

In both zinc oxide-based and resin-based root canal sealers, the use of chitosan and zinc oxide nanoparticles will improve the bactericidal action by preventing the formation of biofilms on the resin-dentin interface. Incorporating bioactive glass, like polyisoprene and polycaprolactone, into polymers rendered the composite bioactive and increased locking ability in a single-rooted tooth’s root canal [25]. Antibacterial nanoparticles enhance efficacy against resistant pathogens in endodontics, which can specifically target bacteria and improve drug delivery in dental applications [26] (e.g., ProTaper, Sendoline S5 system).

Activ Points

Gutta-percha with 5% chlorhexidine diacetate is an Activ point. Here, when chlorhexidine (a good disinfectant) comes into contact with moisture, it releases cations that bind to the cell walls of bacteria, which are anionic, causing osmosis to malfunction.

Resilon

The nano-diamond GP (gutta-percha) composite with amoxicillin was found to have greater mechanical properties than the normal GP.

Smart gels

These gels combine the concept of a solvent-swollen polymer network with the ability to respond to environmental changes. These are used in places where controlled release is needed (e.g., ROS (reactive oxygen species)-responsive hydrogels).

Future potential

Smart materials are transforming dentistry with their innovative properties. They offer potential applications like self-healing fillings, shape-memory alloys for efficient orthodontic treatment, and bioactive materials that promote bone growth. These advancements can lead to better treatment outcomes, reduced costs, and enhanced patient experience, making dentistry effective.

## Conclusions

Smart materials used in dentistry are changing the game, enabling clinicians to provide more effective and efficient care. These materials offer numerous benefits, including enhanced durability and bioactivity, allowing for better treatment outcomes and higher patient satisfaction. By leveraging these innovative materials and staying up to date with the latest evidence-based practices, dental clinicians can take their skills to the next level and deliver truly exceptional care. The future of dentistry is smart, and it is exciting to think about the possibilities.
